# Subchronic Arsenic Exposure Induces Anxiety-Like Behaviors in Normal Mice and Enhances Depression-Like Behaviors in the Chemically Induced Mouse Model of Depression

**DOI:** 10.1155/2015/159015

**Published:** 2015-05-31

**Authors:** Chia-Yu Chang, How-Ran Guo, Wan-Chen Tsai, Kai-Lin Yang, Li-Chuan Lin, Tain-Junn Cheng, Jiunn-Jye Chuu

**Affiliations:** ^1^Department of Neurology, Chi Mei Medical Center, 901 Chung-Hwa Road, Yongkang, Tainan 710, Taiwan; ^2^Department of Biotechnology, College of Engineering, Southern Taiwan University of Science and Technology, 1 Nan-Tai Street, Yongkang, Tainan 710, Taiwan; ^3^Department of Occupational and Environmental Medicine, National Cheng Kung University Hospital, 138 Sheng-Li Road, Tainan 704, Taiwan; ^4^Department of Environmental and Occupational Health, College of Medicine, National Cheng Kung University, 138 Sheng-Li Road, Tainan 704, Taiwan; ^5^Occupational Safety, Health, and Medicine Research Center, National Cheng Kung University, 138 Sheng-Li Road, Tainan 704, Taiwan; ^6^Center for Occupational and Environmental Health and Preventive Medicine, National Cheng Kung University, 138 Sheng-Li Road, Tainan 704, Taiwan; ^7^Department of Occupational Medicine, Chi Mei Medical Center, 901 Chung-Hwa Road, Yongkang, Tainan 710, Taiwan; ^8^Department of Occupational Safety and Health, Institute of Industrial Safety and Disaster Prevention, College of Sustainable Environment, Chia Nan University of Pharmacy and Science, No. 60, Section 1, Erh-Jen Road, Jen-Te, Tainan 711, Taiwan

## Abstract

Accumulating evidence implicates that subchronic arsenic exposure causes cerebral neurodegeneration leading to behavioral disturbances relevant to psychiatric disorders. However, there is still little information regarding the influence of subchronic exposure to arsenic-contaminated drinking water on mood disorders and its underlying mechanisms in the cerebral prefrontal cortex. The aim of this study is to assess the effects of subchronic arsenic exposure (10 mg/LAs2O3 in drinking water) on the anxiety- and depression-like behaviors in normal mice and in the chemically induced mouse model of depression by reserpine pretreatment. Our findings demonstrated that 4 weeks of arsenic exposure enhance anxiety-like behaviors on elevated plus maze (EPM) and open field test (OFT) in normal mice, and 8 weeks of arsenic exposure augment depression-like behaviors on tail suspension test (TST) and forced swimming test (FST) in the reserpine pretreated mice. In summary, in this present study, we demonstrated that subchronic arsenic exposure induces only the anxiety-like behaviors in normal mice and enhances the depression-like behaviors in the reserpine induced mouse model of depression, in which the cerebral prefrontal cortex BDNF-TrkB signaling pathway is involved. We also found that eight weeks of subchronic arsenic exposure are needed to enhance the depression-like behaviors in the mouse model of depression. These findings imply that arsenic could be an enhancer of depressive symptoms for those patients who already had the attribute of depression.

## 1. Introduction

Arsenic is one of the most common environmental contaminants in ground water, and chronic arsenic related health effects are endemic in populations exposed to contaminated water [1]. Chronic arsenic poisoning (arsenicosis) has been shown to increase the risk of neurological disturbances in many epidemiological studies [2–4]. Chronic exposure to inorganic arsenic is associated with neurotoxicity, and inorganic arsenic is usually more harmful than organic arsenic compounds in humans and rodents [5]. Some studies suggest that arsenic can adversely affect brain development and neural function even when present within the permissible limit of arsenic content in drinking water of 0.05 mg/L [6]. Recent animal studies suggest that neurons in the brain may be the major targets of arsenic neurotoxicity and show myelin damage, disappearance of axons, vacuolar degeneration, and loss of cell-cell junction. Arsenic exposure from drinking water containing 1-2 mg/L (approximately 0.1–0.2 mg/kg/day) has been shown to induce oxidative DNA damage in the brain [7–10]. While inorganic arsenic exposure has been correlated with impairments of new learning and recent memory, few studies have explored its effects on mood disorder such as anxiety and depression. Anxiety and depression are currently the most common and well-studied mood disorders in humans and are commonly coexistent [11]. It was proposed that anxiety and depression may be a neuroendocrine continuum, in which anxiety occurs first during the life course and major depressive episodes occurred later [12].

There are hypotheses to explain the mechanisms underlying the depression disorder, including monoamine hypothesis, neurotrophic hypothesis, and neurogenic hypothesis. The deficiency or imbalance in the monoamine neurotransmitters, such as serotonin, dopamine, and norepinephrine, has been shown to be the possible mechanisms underlying the pathophysiology of depression [13]. The 5-HT_1A_ receptor is a key regulator of serotonin activity and its dysregulation is implicated in the emergence of both generalized anxiety disorder and major depression disorder [14]. The 5-HT_1A_ receptor function plays a prominent role in mediating serotonin's postsynaptic receptor mediating serotonin's action on corticolimbic regions including the cerebral frontal cortex, amygdala, and hippocampus [15]. The therapeutic actions and mechanisms of 5-HT_1A_ agonists in alleviating anxiety and depressive disorders have been well documented [16]. Neurotrophic factors are critical signaling molecules for nervous system development, the survival, and adaptive plasticity of neurons in the adult brain; they are also extensively studied in depression [17].

Several lines of evidence suggest that brain-derived neurotrophic factor (BDNF) is involved in depression, such that the expression of BDNF is decreased in depressed patients. In addition, antidepressants upregulate the expression of BDNF. Tropomyosin receptor kinase B (TrkB) is a transmembrane receptor with an intracellular tyrosine kinase domain, which has a high binding affinity for BDNF. These findings have led to the proposal of the “neurotrophin hypothesis of depression” [18–20]. A neurogenic hypothesis of depression has also been postulated, which suggests that reduced adult hippocampal neurogenesis may underlie the pathology of depression. Stress-induced decreases in dentate gyrus neurogenesis are an important causal factor in precipitating episodes of depression. Reciprocally, therapeutic interventions for depression that increase serotonergic neurotransmission act at least in part by augmenting dentate gyrus neurogenesis and thereby promoting recovery from depression [21]. A large body of postmortem and neuroimaging studies of depressed patients has reported reductions in grey-matter volume and glial density in the prefrontal cortex and the hippocampus, mediating the cognitive aspects of depression, such as feelings of worthlessness and guilt [22].

To the best of our knowledge, only a few studies have been conducted in exploring arsenic related mood disorders such as anxiety and depression [23–25]. Furthermore, very little is known about the mechanisms through which arsenic can induce behaviors of mood disorders. In this study, we propose a hypothesis that 4 to 8 weeks of subchronic arsenic exposure (10 mg/L arsenic in drinking water) can induce or enhance anxiety and depression-like behaviors in normal mice and vulnerable mouse model of depression, chemically induced by reserpine pretreatment. Reserpine can induce depression-like behaviors in animals by depletion of catecholamine and blocking reuptake. This animal model has been used for investigating the underlying mechanism of depression [26, 27].

Therefore, the aim of this study is to investigate the anxiety and depression-like behaviors after subchronic arsenic exposure in normal and reserpine pretreated mice and to explore the underlying mechanisms on cerebral prefrontal cortex. After 4 to 8 weeks of subchronic arsenic exposure, the subjects were measured for anxiety-like behaviors by elevated plus maze (EPM) and open field test (OFT) and depression-like behaviors by tail suspension test (TST) and forced swimming test (FST). We also assessed the impact of arsenic exposure on cerebral prefrontal cortex neurodegeneration by measuring immunohistochemistry (IHC) stain of PCNA (proliferating cell nuclear antigen), a marker in early G1 phase and S phase of the cell cycle. To further explore the molecular mechanisms of cerebral prefrontal cortex damage by subchronic arsenic exposure, the levels of the 5-HT/protein kinase A (PKA) signaling-associated proteins (5-HT_1A_ and PKA) and BDNF/TrkB signaling-associated proteins (BDNF, TrkB, and p-Akt) were measured in the cerebral prefrontal cortex of normal and reserpine pretreated mice.

## 2. Materials and Methods

### 2.1. Chemicals and Reagents

As_2_O_3_ was purchased from Sigma-Aldrich (St. Louis, MO, USA). Reserpine was purchased from Acros Organics (Morris Plains, NJ, USA). Mouse monoclonal antibodies against PCNA, 5-HT_1A_ receptor, p-Akt, and *β*-actin were purchased from Santa Cruz Biotechnology Inc. (Dallas, TX, USA). Rabbit polyclonal antibodies against TrkB, CREB, and PKA were purchased from GeneTex, Inc. (Irvine, CA, USA). Nitrocellulose membranes were purchased from NEN Life Science Products Inc. (Boston, MA, USA). Hematoxylin, eosin, xylene, and paraffin were purchased from Thermo Fisher Scientific Inc. (Waltham, MA, USA). Enzyme-linked immunosorbent assay (ELISA) kits were obtained from Promega (Madison, WI, USA).

### 2.2. Animal Experiments

Male C57BL/6 J mice at 5-6 weeks of age (weighing 16–18 g) were purchased from the National Laboratory Animal Center (Taiwan). All animals were maintained in laminar flow cabinets with free access to food and water under specific pathogen-free conditions in facilities approved by the Accreditation of Laboratory Animal Care and in accordance with the Institutional Animal Care and Use Committee (IACUC) of the Animal Research Committee of the Chi-Mei Medical Center (Taiwan). The animals were maintained on a daily 12 h/12 h light/dark cycle, and experiments were carried out between 10:00 h and 17:00 h. After a two-week acclimation period, the mice were randomly segregated into two study populations of 16 each. One study subjects were assigned to either a normal control group (*N* = 8) with drinking water alone or a arsenic exposure group receiving 13.3 *μ*g As_2_O_3_/mL, equal to 10 mg/L arsenic (*N* = 8) through drinking* ad libitum* for 8 consecutive weeks. Another study subjects were pretreated with reserpine for 5 days (2 mg/kg, ip) to create a chemically induced mouse model of depression. Then, they were also assigned to either a reserpine pretreated group with drinking water alone (*N* = 8) or with arsenic exposure (*N* = 8), receiving 10 mg/L As_2_O_3_ through drinking* ad libitum* for 8 consecutive weeks. During the experiments, anxiety- and depression-like behaviors were recorded monthly. At the end of the behavioral experiments (8 weeks later), all tested mice were euthanized for immunohistochemical examination, western blot analysis and enzyme-linked immunosorbent assay (ELISA).

### 2.3. Elevated Plus Maze (EPM)

The EPM test was performed as previously described [28]. The EPM apparatus was constructed from grey Plexiglas and consisted of two open arms and two enclosed arms with 15 cm high transparent walls. The apparatus was elevated to a height of 55 cm above the floor. The mouse was placed in the apparatus to start exploring the maze from the central square of the apparatus (facing an enclosed arm) for 10 min. The total time spent in the open and closed arms and the total entries were measured. For data analysis, the ratio of entries into open arms to closed arms was analyzed for the evaluation of anxiolytic activity. Data acquisition and analysis were performed automatically using Image EP software (O'Hara & Co., UK).

### 2.4. Open Field Test

To analyze anxiety related behavior, ambulatory activity was measured using the open field test as previously described [29]. Mice were allowed to adapt to the environment for one hour prior to testing. Mice were placed individually in the center of a square open field (50 × 50 × 50 cm) with white Plexiglas walls and were observed for 10 minutes under normal lighting. Movements and trajectories of mice were videotaped and analyzed by the TM-01 Animal Video Behavior System (Diagnostic & Research Instruments Co. Taiwan), which is a versatile video tracking system for automatically recording and analyzing animal activity, movement, and behavior. All data of given parameters, such as motion tracking trajectory, total ambulatory time, and % ratio of the time spent in all four 10 × 10 cm square corners were recorded, respectively, and were calculated by the same TM-01 Video Tracking Software program.

### 2.5. Tail Suspension Test (TST)

The TST was performed as described previously [30]. Mice were tested for 10 min, and the cumulative immobility time (latency to immobility, number of immobile segments, and total time spent immobile) during the final 8 min interval of the test was recorded. The total duration of immobility (in seconds) was measured during the 8 min period. A decrease in the duration of immobility was indicative of an antidepressant-like effect.

### 2.6. Forced Swimming Test (FST)

Individual mice were forced to swim in an open cylindrical container (25 cm height × 10 cm diameter) filled with water at 24.5–25.5°C up to a height of 10 cm. In our study, the total duration of immobility was evaluated in a 5 min swim session in the FST, which was modified on the basis of a previous study [31]. The mice were considered immobile when they made only the movements necessary to keep their head above water. The test was performed at 1 h after the last treatment, and groups of mice were tested in parallel. Each test was conducted in a quiet and warm environment. A decrease in the duration of immobility was indicative of an antidepressant-like effect.

### 2.7. Immunohistochemistry (IHC) Staining

Following termination, the tested mice were anaesthetized with a lethal dose of pentobarbital and perfused transcardially with PBS followed by ice cold 4% paraformaldehyde. The intact cerebral prefrontal cortex was excised and postfixed for 24–72 h in 4% paraformaldehyde at 4°C. Samples were dehydrated by passing them through a gradient mixture of ethyl alcohol and water. They were then rinsed with xylene and embedded in paraffin. The formalin fixed tissues were sliced into 5 mm sections on silane-coated slides using a Microtome RM2135 (Leica Microsystems Inc., Bannockburn, IL), rehydrated in graded ethanol solutions, immersed in tris-buffered saline (TBS, pH 7.4), dried overnight at 37°C, and stored at room temperature until use. For immunocytochemistry, the sections were stained as previously described [32] to identify cerebral cortical degenerating neurons. The sections were soaked in 0.3% hydrogen peroxide in methanol for 20 min to remove endogenous peroxidase activity, rinsed in Tris-buffered saline (TBS), and pretreated in a solution of 0.1 M citrate buffer heated to 90–95°C for 10 min. Sections were cooled and rinsed in TBS. The sections were incubated with Block Ace (DS Pharma Biomedical, Osaka, Japan) for 1 h at room temperature, to prevent nonspecific binding of immunoglobulin. Tissue sections then were incubated overnight with primary antibodies against PCNA (proliferating cell nuclear antigen), a marker for cells in early G1 phase and S phase of the cell cycle. After overnight incubation with the primary antibody (1 : 200), the sections were washed three times in TBS before applying the secondary antibody. The sections were incubated with biotinylated goat anti-rabbit IgG (1 : 500) for 30 min at 37°C, followed by incubation with horseradish peroxidase-conjugated streptavidin (1 : 500) for 30 min at room temperature. Immunoreactivity was visualized by treating sections with DAB (3,3′-diaminobenzidine) as a substrate for visualization of the Chromagen. Some sections were counterstained with hematoxylin to aid in cell count studies, and all sections were covered with Permount.

### 2.8. Western Blot Analysis

The fresh cerebral PFC was weighed and frozen at −80°C. For western blot analysis, the tissues were prepared in lysis buffer (1% NP-40, 150 mM NaCl, 20 mM Tris-HCl pH 7.5, and protease inhibitors). After incubation on ice for 30 minutes, the lysates were centrifuged (Centrifuge 5804R; Eppendorf Co. Ltd., USA) at 13,000 g for 30 minutes. Postnuclear supernatants were mixed with equal volumes of 2x sample buffer (12.5 mm Tris-HCl pH 6.8, 2% SDS, 20% glycerol, and 0.25% bromophenol blue) and boiled for 5 minutes. The samples were separated in 10% polyacrylamide gels and then transferred to nitrocellulose membranes. The membranes were blocked with bovine serum albumin in 0.1 M phosphate buffer saline with Tween (0.2 M Na2HPO4, 0.2 M NaH2PO4, 1.5 M NaCl, and 0.1% Tween 20) for 2 hours at room temperature. After blocking, the membranes were incubated with the primary anti-5-HT_1A_ antibody, anti-PKA antibody, and anti-TrkB antibody at a 1/500 dilution, and primary anti-pAkt antibody at a 1/1000 dilution in wash buffer for 1 hour at room temperature, followed by four 10-minute washes. The membranes were then incubated with a horseradish peroxidase-conjugated anti-rabbit IgG antibody diluted 1 : 5,000 in wash buffer for 1 hour at room temperature, and horseradish peroxidase was added to visualize antibody-bound target protein on the nitrocellulose membrane. Western blot images were obtained using a LAS-3000 analyzer (FUJIFILM LAS-3000; Fuji Photo Film Co. Ltd., Japan). The signals for *β*-actin were also evaluated to normalize protein loading.

### 2.9. Enzyme-Linked Immunosorbent Assay (ELISA)

The fresh cerebral PFC was weighed and frozen at −80°C and homogenized at a 1 : 30 (wt/vol) dilution in a high salt extraction buffer (100 mM Tris-HCl (pH 7.6), 1 M NaCl, 2% BSA, 4 mM EDTA, and 0.5% Triton X-100) and supplemented with protease inhibitors. After centrifugation at 6,500 g for 30 minutes at room temperature, the supernatants were stored at −80°C until assayed. The blood was also prepared by centrifuging at 1450 g for 10 minutes at 4°C. Serum was separated and stored at −80°C for subsequent analyses. Total protein assay was performed using the BCA protein assay kit, according to the Bradford method provided by the manufacturer. The BDNF content was assayed using the ChemiKine BDNF Sandwich ELISA (Chemicon, Temecula, CA) and assessed for color intensity with a microplate reader at 450 nm. BDNF concentrations were calculated through interpolating absorbance readings from standard curves generated with calibrated BDNF protein standards provided by the manufacturer. All reagents, dilutions, and calculations were applied according to the manufacturer's instructions.

### 2.10. Statistical Analysis

All results were presented as the mean ± standard deviation (SD). Differences between groups were evaluated by analysis of variance and post* hoc* comparisons with the Bonferroni step-down (Holm) correction. Statistical analysis was performed using SigmaPlot software (version 10.0; SPSS Inc., USA). Post hoc testing of behavioral data utilized a two-tailed Welch's *t*-test. Post hoc testing of biochemical data utilized a regression analysis. Each value represents the mean ± SD of 8 mice. *P* values less than 0.05 were considered statistically significant differences. ^*∗*^ *P* < 0.05, ^*∗∗*^ *P* < 0.01, and ^*∗∗∗*^ *P* < 0.001 represent significant differences from the normal group (drinking water alone). ^#^ *P* < 0.05, ^##^ *P* < 0.01 represent significant differences from the ES/R group.

## 3. Results

### 3.1. Subchronic Arsenic Exposure Induces Only Anxiety-Like Behaviors in Normal Mice

**Figure 1:**
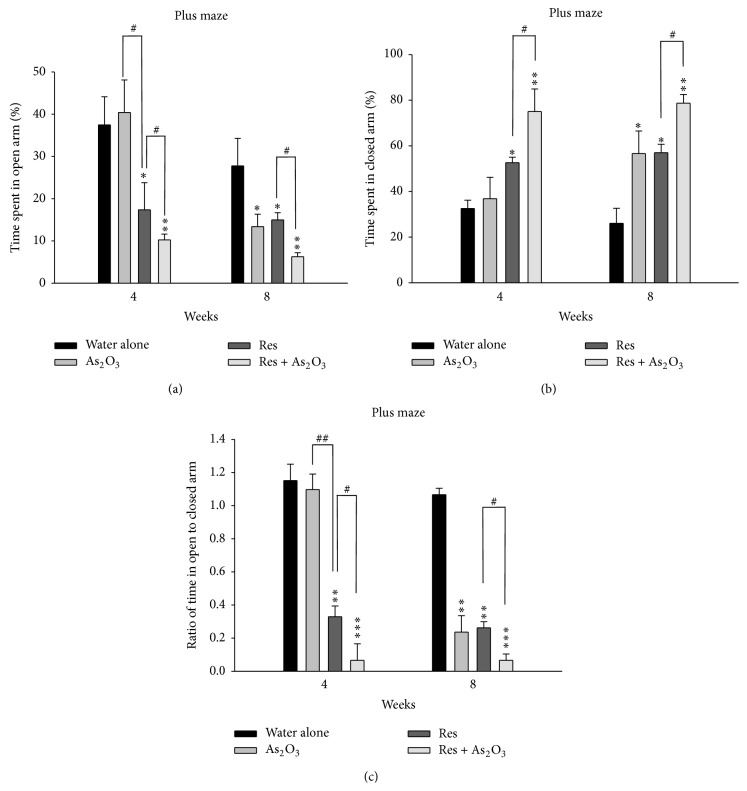
The effects of subchronic arsenic exposure on EPM behavior test. Normal mice and reserpine pretreated mice treated with 10 mg/L arsenic or drinking water alone were analyzed by the elevated plus maze (EPM) test at 4 and 8 weeks. The percentage of time spent (as 100% in 10 minutes) in the open arms (a) and closed arms (b) and the % ratio of time spent in the open/closed arms (c) were evaluated during the 10-minute test period. Each value/error bar is expressed as the mean ± SD of 8 mice. ^*∗*^ *P* < 0.05, ^*∗∗*^ *P* < 0.01, and ^*∗∗∗*^ *P* < 0.001, significant difference compared with normal control group. ^#^ *P* < 0.05, ^##^ *P* < 0.01, significant difference from the reserpine group (Res).

**Figure 2:**
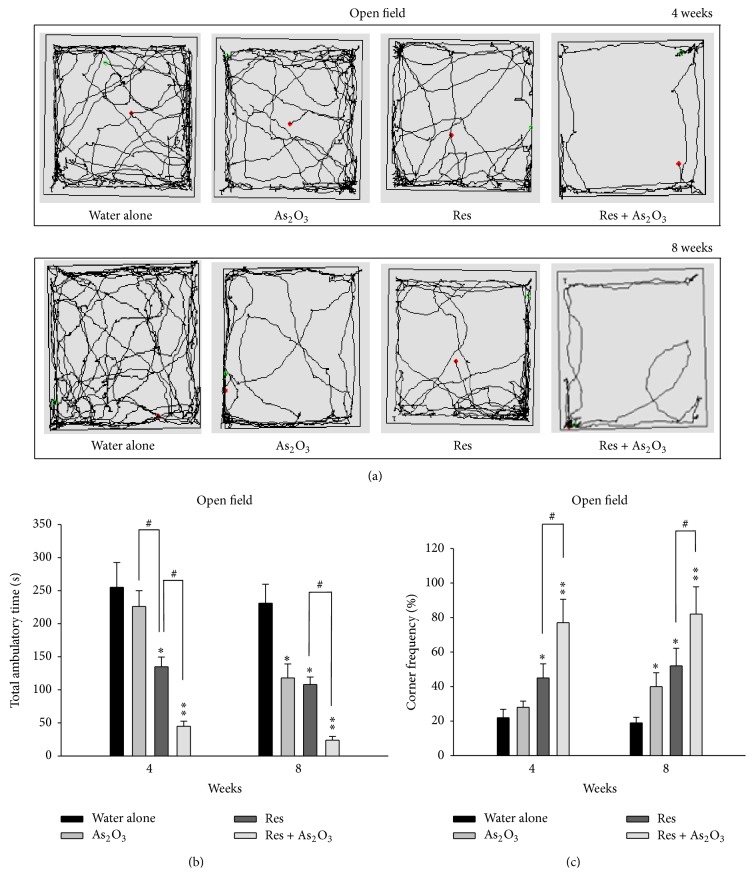
The effects of subchronic arsenic exposure on exploratory behavior. Normal mice and reserpine pretreated mice treated with 10 mg/L arsenic or drinking water alone were analyzed by the open field test (OFT) at 4 and 8 weeks. Each mouse was placed in a corner of an “open field,” allowed to roam the field and was automatically recorded within 10 minutes by a TM-01 Animal Video Behavior System. The representative images (motion tracking behavior) showing the mouse traveling patterns are shown in (a). Exploratory behaviors, including total ambulatory time (b) and % ratio of the time spent in all four corner zones (c), were measured during 10 minutes in mice. Each value/error bar is expressed as the mean ± SD of 8 mice. ^*∗*^ *P* < 0.05, ^*∗∗*^ *P* < 0.01, significant difference compared with normal control group. ^#^ *P* < 0.05, significant difference from the reserpine group (Res).

**Figure 3:**
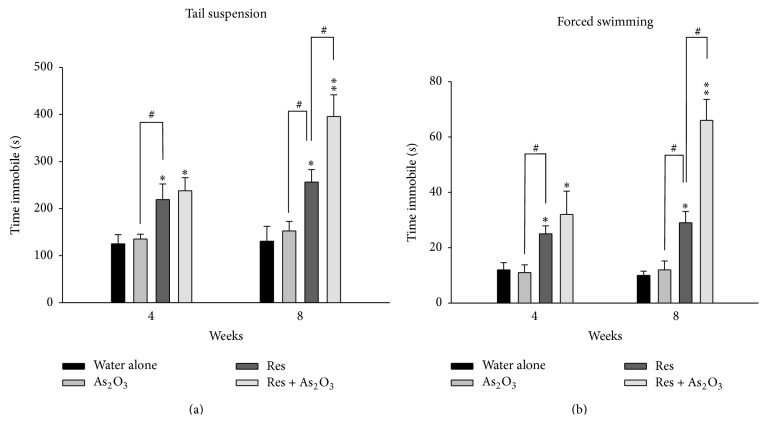
The effects of subchronic arsenic exposure on TST and FST behavior tests. Normal mice and reserpine pretreated mice treated with 10 mg/L arsenic or drinking water alone were analyzed by the tail suspension test (TST) and forced swimming test (FST) at 4 and 8 weeks. The total duration of immobility (in seconds) in the tested mice was measured on the TST (a) during the 8-minute period and FST (b) in a 5-minute swim session, alternatively. Each value/error bar is expressed as the mean ± SD of 8 mice. ^*∗*^ *P* < 0.05, ^*∗∗*^ *P* < 0.01, significant difference compared with normal control group. ^#^ *P* < 0.05, significant difference from the reserpine group (Res).

We exposed the experimental mice to an arsenic level in drinking water of 10 mg/L of arsenic As_2_O_3_, comparable to the mean toxic dose in previous animal studies [33–35]. After 4 weeks of As_2_O_3_ exposure, the normal mice showed no significant change in time spent in the open/closed arms (^*∗*^ *P* > 0.05, ^*∗*^ *P* > 0.05, resp.) and the ratios of entries into open arms to closed arms (^*∗*^ *P* > 0.05). However, after 8 weeks, they showed decreased time spent (^*∗*^ *P* < 0.05) in the open arms (Figure 1(a)), increased time spent (^*∗*^ *P* < 0.05) in the closed arms (Figure 1(b)), and reduced the ratios of entries (^*∗*^ *P* < 0.01) into open arms to closed arms (Figure 1(c)). Similarly, in the open field test, the motion tracking analysis demonstrated that the normal mice with As_2_O_3_ exposure had less motion tracking (illustrated by the black line) (Figure 2(a)) and significantly decreased total ambulatory time (^*∗*^ *P* < 0.05) and increased time spent (^*∗*^ *P* < 0.05) in all four corner zones (% corner frequency) only after 8 weeks of arsenic exposure (Figures 2(b) and 2(c)). In contrast, in the tests for the depression-like behaviors, the normal mice after 4 weeks or 8 weeks of As_2_O_3_ exposure did not show significant difference in the TST and FST for duration of immobility (Figure 3).

### 3.2. Subchronic Arsenic Exposure Enhances Both Anxiety and Depression-Like Behaviors in the Reserpine Induced Mouse Model of Depression

In this study, we create a chemically induced mouse model of depression by pretreatment with reserpine (2 mg/kg, ip) for 5 days. For data analysis, decreased time spent in the open arms, lengthened time spent in the closed arms, and the elevated the ratio of entries into open arms to closed arms would be highly regarded as anxiety induction. As expected, the reserpine pretreated mice induced obvious anxiety-like behaviors observed after 4 weeks and 8 weeks. In the EPM test, these mice showed decreased time spent (^*∗*^ *P* < 0.05, ^*∗*^ *P* < 0.05, resp.) in the open arms (Figure 1(a)), prolonged time spent (^*∗*^ *P* < 0.05, ^*∗*^ *P* < 0.05, resp.) in the closed arms (Figure 1(b)), and reduced ratios of entries (^*∗*^ *P* < 0.01, ^*∗*^ *P* < 0.01, resp.) into open arms to closed arms (Figure 1(c)) at 4 weeks and 8 weeks. Furthermore, after 4 and 8 weeks of As_2_O_3_ exposure, all the reserpine pretreated mice showed enhanced anxiety-like behavior with significantly decreased time spent (^#^ *P* < 0.05) in the open arms (Figure 1(a)), increased time spent (^#^ *P* < 0.05) in the closed arms (Figure 1(b)), and reduced ratios of entries (^#^ *P* < 0.05) into open arms to closed arms (Figure 1(c)).

Similarly, in open field test, they also showed markedly reduced locomotion activity and greater distribution of traveling tracts across surrounding exposure (Figure 2(a)), and significantly decreased total ambulatory time (^#^ *P* < 0.05) (Figure 2(b)) and increased corner frequency (^#^ *P* < 0.05) (Figure 2(c)). As our expectation, the reserpine pretreated mice also showed obvious depression-like behavior in TST and FST at 4 weeks (^*∗*^ *P* < 0.05, ^*∗*^ *P* < 0.05, resp.) and 8 weeks (^*∗*^ *P* < 0.05, ^*∗*^ *P* < 0.05, resp.) when compared to the normal control mice (Figures 3(a) and 3(b)). Furthermore, after 8 weeks of As_2_O_3_ exposure, the reserpine pretreated mice also showed exacerbated depression-like behavior with increased immobility period of tail suspension (^#^ *P* < 0.05) and swimming performances (^#^ *P* < 0.05) (Figures 3(a) and 3(b)).

### 3.3. Subchronic Arsenic Exposure Interfere with Cell Proliferation on Cerebral Prefrontal Cortex

Previous studies in arsenite-treated cultured primary rat hippocampal neurons showed symptoms of apoptosis such as decreased viable cell growth, cytoplasm vacuoles, frothing, and nuclear condensation with intact membrane [36]. However, our previous data did not find reduction of cell number and size in the CA1 and dentate gyrus (DG) regions of hippocampal sections (Hematoxylin and eosin stain) either in normal mice or in reserpine pretreated mice after daily drinking water of 10 mg/L As_2_O_3_ for 8 weeks (data not shown). PCNA (proliferating cell nuclear antigen) is abundant in actively proliferating cells in S phase of the cell cycle and in nondividing cells undergoing DNA synthesis and repair [37]. By PCNA stains for qualitative assessment of PCNA-positive cells, we studied whether subchronic 8 weeks of As_2_O_3_ exposure leads to detriments on cerebral prefrontal cortex in normal mice and reserpine pretreated mice.

**Figure 4:**
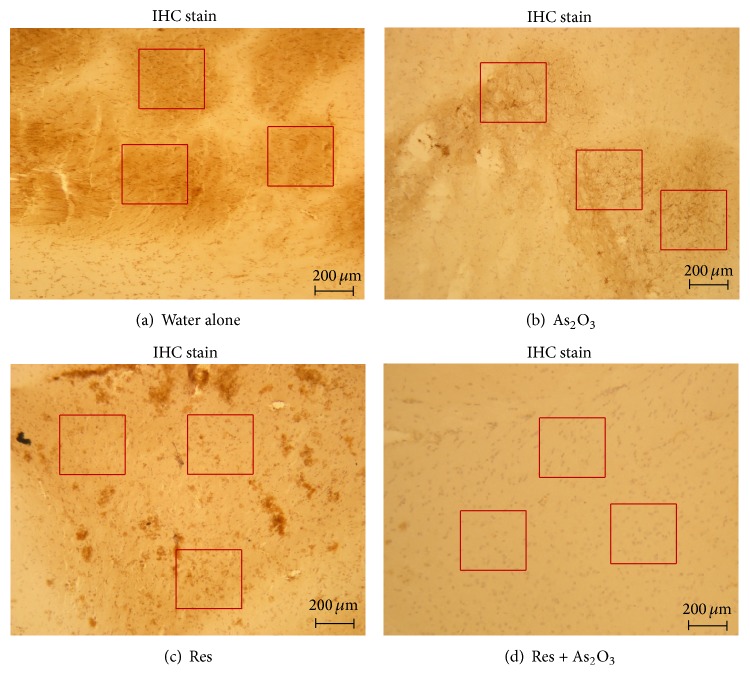
The influence of subchronic arsenic exposure on cell proliferation of cerebral PFC neuron. Cerebral PFC was isolated and studied for histopathology 8 weeks after treatment with drinking water alone (a) and 10 mg/L arsenic (b) in normal mice and drinking water alone (c) and 10 mg/L arsenic (d) in reserpine pretreated mice. The representative staining photomicrographs of PCNA-positive cells in each section were examined (magnification ×40) under light microscopy (scale bar = 200 *μ*m). After 5 random fields from each section were captured, the number of intensely PCNA-positive cells (in brown) in corresponding proliferative zone (including the ventricular and subventricular zones) within each field was computed by using Image Pro Plus software (Version 5.1) and was compared to the total number of cells.

Photomicrographs of the cerebral prefrontal cortex (IHC stain) showed intact structural integrity in all tested groups (Figure 4). The data showed that the number of PCNA-positive cells was 32.5 ± 2.9 in the normal control mice, 16.5 ± 1.4 in the normal mice with As_2_O_3_ exposure (^*∗*^ *P* < 0.05), 12.0 ± 2.6 in the reserpine pretreated mice (^*∗*^ *P* < 0.05), and 3.6 ± 0.5 in the reserpine pretreated mice with As_2_O_3_ exposure (^#^ *P* < 0.05) in each 300 × 300 *μ*m field (red square). Compared to normal controls (Figure 4(a)), there was a significant 48.5 ± 10.8% reduction of the PCNA-positive cells in the normal mice with As_2_O_3_ exposure (Figure 4(b)) and 65.8 ± 16.2% reduction in the reserpine pretreated mice (Figure 4(c)). Similarly, the reserpine pretreated mice with As_2_O_3_ exposure (Figure 4(d)) showed a significant 70.2 ± 20.5% reduction of the PCNA-positive cells when compared to those without As_2_O_3_ exposure.

### 3.4. Subchronic Arsenic Exposure Enhances Depression-Like Behaviors Possibly through BDNF/TrkB/p-Akt Pathways in Reserpine Pretreated Mice

**Figure 5:**
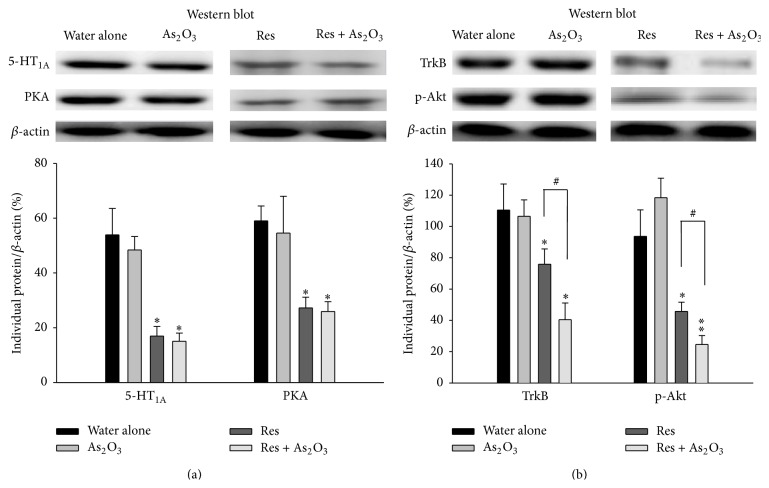
The influence of subchronic arsenic exposure on the expression levels of 5-HT_1A_ receptor, PKA, TrkB, and p-Akt in the cerebral PFC. Eight weeks after exposure with 10 mg/L arsenic or drinking water alone in normal mice and in reserpine pretreated mice, the expression levels of 5-HT_1A_ receptor and PKA proteins (a) and the expression levels of TrkB, p-Akt (b) in the cerebral PFC of the tested mice were detected by western blot analysis. These protein expression levels were quantitated by microcomputer image device (MCID) image analysis. The lower panel shows the individual protein/*β*-actin ratios while the *β*-actin levels were evaluated as a loading control. Each value/error bar is expressed as the mean ± SD of 8 mice.^*∗*^ *P* < 0.05, ^*∗∗*^ *P* < 0.01, significant difference compared with normal control group (drinking water alone). ^#^ *P* < 0.05, significant difference from the reserpine group (Res).

Prior research has shown that inhibited expression of cerebral 5-HT_1A_ receptor may contribute to a behavioral phenotype of anxiety and depression in rodents [38]. We therefore measured the levels of 5-HT_1A_ receptor and its downstream target PKA in cerebral prefrontal cortex of normal mice or reserpine pretreated mice by western blot after 8 weeks of arsenic exposure. We did not find any differences in the levels of 5-HT_1A_ receptor and PKA in normal mice with or without As_2_O_3_ exposure. But, as expected, marked decreased levels of 5-HT_1A_ receptor and PKA were found in cerebral prefrontal cortex of reserpine pretreated mice (^*∗*^ *P* < 0.05, ^*∗*^ *P* < 0.05, resp.). Nevertheless, As_2_O_3_ exposure did not further inhibit the levels of 5-HT_1A_ receptor and PKA in reserpine pretreated mice (^#^ *P* > 0.05, ^#^ *P* > 0.05, resp.) (Figure 5(a)). In measuring the downstream signals of BDNF-TrkB pathway, reduced cerebral prefrontal cortex protein levels of TrkB and the ratio of p-Akt/*β*-actin were found in reserpine pretreated mice when compared with the normal controls. However, As_2_O_3_ exposure in reserpine pretreated mice significantly exacerbated the inhibitory effect of reserpine in cerebral prefrontal cortex protein levels of TrkB and the ratio of p-Akt/*β*-actin (^#^ *P* < 0.05, ^#^ *P* < 0.05, resp.) (Figure 5(b)).

**Figure 6:**
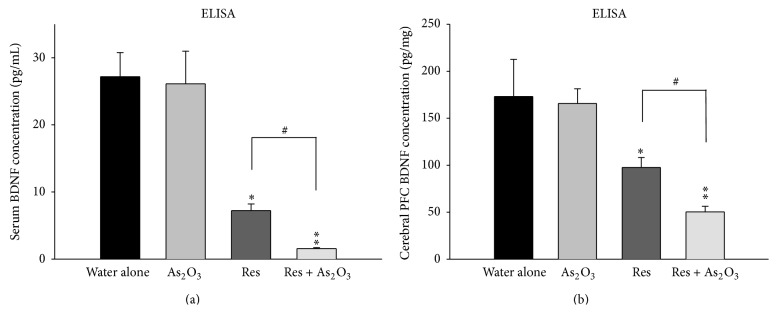
The influence of subchronic arsenic exposure on the BDNF levels of serum and cerebral PFC. Eight weeks after exposure to 10 mg/L arsenic or drinking water alone in normal mice and in reserpine pretreated mice, the levels of BDNF protein of serum (a) and cerebral PFC (b) were determined by enzyme-linked immunosorbent assay (ELISA). Each value/error bar is expressed as the mean ± SD of 8 mice. ^*∗*^ *P* < 0.05, ^*∗∗*^ *P* < 0.01, significant difference compared with normal control group.

We then investigated whether subchronic arsenic exposure could induce or enhance anxiety- or depression-like behaviors through downregulation of BDNF-TrkB signaling pathway. After 8 weeks of As_2_O_3_ exposure, the BDNF levels of serum (Figure 6(a)) and cerebral prefrontal cortex (Figure 6(b)) were not changed in normal mice. In contrast, exacerbated inhibitory effect of reserpine in the BDNF levels of serum (^*∗*^ *P* < 0.05) and cerebral prefrontal cortex (^*∗*^ *P* < 0.05) were found in the reserpine pretreated mice (Figures 6(a) and 6(b)). Moreover, in accordance with the above findings of TrkB/p-Akt expression, As_2_O_3_ exposure in reserpine pretreated mice also significantly reinforce the inhibitory effect of reserpine in the BDNF levels of serum (^#^ *P* < 0.05) and cerebral prefrontal cortex (^#^ *P* < 0.05) (Figures 6(a) and 6(b)).

## 4. Discussion

In this present study, we demonstrated that subchronic arsenic exposure induces anxiety-like behaviors in normal mice and enhances depression-like behaviors in the chemically induced mouse model of depression. After 8 weeks of arsenic exposure, the normal mice showed prominent anxiety-like behaviors in all the behavior tests for anxiety (elevated plus maze and open field test) (Figures 1 and 2). We created a chemically induced mouse model of depression by pretreatment with reserpine (2 mg/kg, ip) for 5 days. As expected, the reserpine pretreated mice showed obvious anxiety- and depression-like behaviors in the elevated plus maze, open field, tail suspension, and forced swimming tests and these behaviors were enhanced after 4 and 8 weeks of arsenic exposure (Figures 1, 2, and 3).

Clinical studies on psychiatric comorbidities have demonstrated that arsenicosis patients show psychiatric ailments such as depression, mixed anxiety, and depressive disorder [4]. In animal studies, perinatal exposure to relatively low levels of arsenic (0.05 mg/L) also significantly increase learned helplessness and immobility in a forced swim task that predisposes affected offspring to depressive-like behavior in the affected adult C57BL/6J mouse model [39]. It has been reported that 14-day exposure of arsenic trioxide increased the metabolites of 5-hydroxytryptamine, norepinephrine, and dopamine in the cerebral cortex, hippocampus, and hypothalamus, resulted in changes in the vertical and horizontal motor activity in mice [40]. After 5–20 mg/kg of arsenic exposure for duration of 2–4 weeks, the reduced locomotor activity and the alterations in monoamine content with increasing arsenic brain concentration were observed in the midbrain and cortex of rat [41]. Several studies further indicated that NaAsO2 impairs neuritogenesis with the suppression of GluA1 expression in primary cultures of neurons obtained from the cerebral cortex of mouse [42] and induce oxidative stress (lipid peroxidation) and sphingolipidosis in various rat brain regions, including cerebral cortex [43]. Pathological reports also indicate degenerative changes in the cerebral cortex, such as loss of synaptic vesicles, accompanying decreases of norepinephrine, dopamine, and 5-hydroxytryptamine (5-HT) in the striatum, hippocampus, and other cerebral regions in both mice and rats after exposure to arsenic exceeding permitted levels [44].

Here, we studied the effects of subchronic arsenic exposure (4–8 weeks) on normal mice and the mouse model of depression induced chemically by reserpine pretreatment. Reserpine is an antihypertensive drug that irreversibly inhibits the vesicular uptake of monoamine neurotransmitters, including norepinephrine, dopamine, and 5-hydroxytryptamine (5-HT), producing a clinically significant depression-like state [45]. Reserpine also dose-dependently increases anxiety related behavior in mice (decreased locomotion and exploration) at dosages of 0.4 to 1.6 mg/kg [46]. Rats treated with reserpine also showed significantly lowered levels of horizontal locomotion and vertical exploration [47]. In this study, although 8 weeks of subchronic arsenic exposure (10 mg/L As_2_O_3_ in drinking water) showed no significant depression-like behavior in TST and FST in normal mice, it indeed induces anxiety-like behaviors. In addition, although no significant anxiety-like behaviors were shown in 4 weeks of subchronic arsenic exposure in normal mice, this duration is sufficient to enhance the anxiety behavior in the reserpine pretreated mice. However, enhancing the depression-like behavior 8 weeks of subchronic arsenic exposure is needed. Our results are compatible with the previous notation that anxiety and depression may be a neuroendocrine continuum, in which anxiety occurs first during the life course and major depressive episodes occur later [12]. Despite the fact that arsenic could be an enhancer of depressive symptoms for those patients who already had the attribute of depression, a reduction in the immobility time in the forced swimming test and tail suspension test may be at least partly due to the reduction in the locomotor activity of mice.

Previous studies in arsenite-treated cultured primary rat hippocampal neurons showed symptoms of apoptosis such as decreased viable cell growth, cytoplasm vacuoles, frothing, and nuclear condensation with intact membrane [36]. Pathological reports also indicate degenerative changes in the cerebral cortex, such as loss of synaptic vesicles, accompanying decreases of norepinephrine, dopamine, and 5-hydroxytryptamine (5-HT) in the striatum, hippocampus, and other cerebral regions in both mice and rats after exposure to arsenic exceeding permitted levels [44]. However, our previous data did not find, either in normal mice or in reserpine pretreated mice, a reduction of cell number and size in the CA1 and dentate gyrus (DG) regions of hippocampal sections (H&E stain) after daily drinking water of 10 mg/L As_2_O_3_ for 8 weeks (data not shown). The cerebral cortex, in particular the medial prefrontal cortices (mPFC), and the ventromedial prefrontal cortex (vmPFC), have been most consistently linked to anxious/depressed symptoms [48]. In addition, depressed people display abnormally low activity in the prefrontal cortex in brain-imaging studies using single photon emission tomography (SPECT), positron emission tomography (PET), and magnetic resonance imaging (MRI) [49]. The prefrontal cortex is known not only to be involved in emotional responses, but also to have numerous connections with other parts of the brain that are responsible for controlling dopamine, norepinephrine, and serotonin, three neurotransmitters that are important in mood regulation [50]. In the present study, we therefore measured the impact of arsenic exposure on cerebral prefrontal cortex by measuring immunohistochemistry stain of PCNA, a marker in early G1 phase and S phase of the cell cycle. We demonstrated that there was a significant reduction of the PCNA-positive cells after subchronic arsenic exposure or reserpine pretreatment in the normal mice and a further reduction was also found in reserpine pretreated mice after arsenic exposure (Figure 4). These data might suggest that cerebral prefrontal cortex rather than hippocampus might be one of the major targets after subchronic arsenic intoxication. In accordance with our findings, it was noted in previous studies that inhibition of hippocampus cell proliferation and neurogenesis by 4.0 mg/L As_2_O_3_ for 2 months is reversible upon termination of arsenic administration [23] and that the 8-hydroxy-2′-deoxyguanosine (8-OHdG) levels were significantly increased in degenerative cerebral cortex neurons after mice fed with drinking water containing 1-2 mg/L arsenic [7].

To the best of our knowledge, only a few studies have been conducted in exploring arsenic related mood disorders such as anxiety and depression and very little is known about the mechanisms through which arsenic can induce behaviors of mood disorders. In this present study, it was found that arsenic exposure did not influence 5-HT_1A_ receptor and PKA expressions in cerebral prefrontal cortex of normal mice and reserpine pretreated mice, suggesting that 5-HT_1A_/PKA signal transduction may not be the major target for arsenic intoxication in cerebral prefrontal cortex. Although we did not find decreased BDNF levels in serum and cerebral prefrontal cortex of normal mice after subchronic arsenic exposure, which presented the behaviors of anxiety, we did find decreased BDNF levels in serum and cerebral prefrontal cortex of reserpine pretreated mice, which presented the behavior of depression (Figure 6). This finding is consistent with previous study which showed that forebrain-specific BDNF knockout (CaMK-BDNF (KO)) mice had no anxiety behavior, but increased depression behavior [51]. In parallel, reduced cerebral prefrontal cortex protein levels of TrkB and p-Akt and the downstream signals of BDNF-TrkB pathway were found in reserpine pretreated mice, suggesting that BDNF-TrkB signal pathway on cerebral prefrontal cortex may play a major neurobiological role in depression behaviors induced by reserpine. Furthermore, the reserpine pretreated mice after exposed to arsenic subchronically showed exacerbated inhibition of the BDNF-TrkB signal pathway, which corresponded with consequent enhanced depression-like behaviors.

In summary, in this present study, we demonstrated that subchronic arsenic exposure induces only the anxiety-like behaviors in normal mice and enhances the depression-like behaviors in the reserpine induced mouse model of depression, in which the cerebral prefrontal cortex BDNF-TrkB signaling pathway is involved. We also found that eight weeks of subchronic arsenic exposure is needed to enhance the depression-like behaviors in the mouse model of depression. These findings imply that arsenic could be an enhancer of depressive symptoms for those patients who already had the attribute of depression.
